# Supplementary material for: Effectiveness and Safety of Ayurvedic Medicines in Type 2 Diabetes Mellitus Management: A Systematic Review and Meta-Analysis
Source: Front Pharmacol. 2022 Jun 8;13:821810. doi: 10.3389/fphar.2022.821810 (PMC9213670; doi:10.3389/fphar.2022.821810)
Supplement: Supplementary file 1 [file DataSheet1.zip › Supplementary Material/Forest and Funnel Plots/Nigella sativa/Fasting insulin.pdf]

Nigella sativa - Fasting insulin

Study	Intervention N	Intervention Mean	Intervention SD	Comparator N	Comparator Mean	Comparator SD	Mean Diff. with 95% CI	Weight (%)
Heshmati 2015	36	11.00	3.30	36	13.70	4.60	-2.70 [-4.55, -0.85]	48.91
Jangjo-Borazjani 2021	20	7.86	3.16	20	8.76	2.53	-0.89 [-2.66, 0.88]	51.09
**Overall**							-1.78 [-3.55, -0.00]	

Heterogeneity: $\tau^2 = 0.78$, $I^2 = 47.83\%$, $H^2 = 1.92$

Test of $\theta_i = \theta_j$: Q(1) = 1.92, p = 0.17

Test of $\theta = 0$: z = -1.96, p = 0.05

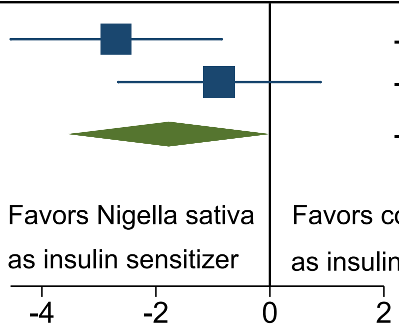

Random-effects REML model
